# A case report of *Coccidioides posadasii* meningoencephalitis in an immunocompetent host

**DOI:** 10.1186/s12879-019-4329-0

**Published:** 2019-08-16

**Authors:** Raynell Lang, William Stokes, Jane Lemaire, Andrew Johnson, John Conly

**Affiliations:** 1 Department of Medicine, Cumming School of Medicine, University of Calgary and Alberta Health Services, Calgary, AB Canada; 2 Departments of Medicine, Microbiology, immunology & Infectious Diseases, Pathology & Laboratory Medicine. Cumming School of Medicine, University of Calgary and Alberta Health Services, Calgary, AB Canada

**Keywords:** Coccidiomycosis, *Coccidioides posadasii*, Meningoencephalitis, Canada

## Abstract

**Background:**

*Coccidioides* spp*.* are dimorphic fungi endemic to Central America, regions of South America and southwestern USA. Two species cause most human disease: *Coccidioides immitis* (primarily California isolates) and *Coccidioides posadasii*. Coccidioidomycosis is typically acquired through inhalation of soil or dust containing spores. Coccidioidal meningitis (CM), most common in the immunocompromised host, can also affect immunocompetent hosts.

**Case presentation:**

We report a case of *C. posadasii* meningoencephalitis in a previously healthy 42-year-old Caucasian male who returned to Canada after spending time working in New Mexico. He presented with a 3-week history of headache, malaise and low-grade fevers. He developed progressive confusion and decreasing level of consciousness following hospitalization. Evidence of hydrocephalus and leptomeningeal enhancement was demonstrated on magnetic resonance imaging (MRI) of his brain. Serologic and PCR testing of the patient's CSF confirmed *Coccidioides posadasii*. Despite appropriate antifungal therapy he continues to have significant short-term memory deficits and has not returned to his full baseline functional status.

**Conclusions:**

Travel to endemic regions can result in disease secondary to *Coccidioides* spp*.* and requires physicians in non-endemic areas to have a high index of suspicion. Effective therapeutic options have reduced the mortality rate of CM, however, it is still associated with significant morbidity and requires life-long therapy.

## Background

*Coccidioides* species are dimorphic fungi within the Ascomycete division [1]. The two species that have been found to cause human disease are *Coccidioides immitis* and *Coccidioides posadasii* [2, 3]. *C. immitis* and *C. posadasii* are morphologically identical with no known phenotypic differences in pathogenicity. These fungi are commonly found in the environment, in the soil of hot and arid ecosystems. The largest difference between the two species is their geographic distribution with *C. immitis* being found predominantly in California and *C. posadasii* in Nevada, Arizona, New Mexico, Texas, Central and South America [2–6]. Serological testing cannot distinguish the two species, they are differentiated only by genetic polymorphisms and subtle differences in mycelial growth characteristics [1, 6].

Both species have a saprophytic and parasitic life cycle. During the saprophytic phase, the fungus lives in soil where mycelia feed off organic material in the environment. When conditions become harsh, the mycelia produce highly resistant spores, called arthroconidia. Arthroconidia can remain viable in soil for years and can be released into the air through soil disruption [1, 5]. Inhalation of arthroconidia leads to infection by conversion into spherules within the susceptible host. The spherules rupture, releasing endospores into surrounding tissues, producing more spherules [1, 5]. If cultured, the spherules revert to mycelia [1, 2, 7].

The most common mode of acquisition is through inhalation of spores. Rarely, transmission occurs through solid organ transplantation or direct inoculation via penetration of skin by contaminated objects [5, 7–10]. While the majority of infected individuals are asymptomatic, symptomatic cases of coccidiomycosis present as mild flu-like symptoms, muscle and joint pain, rash and pulmonary symptoms [4, 5]. Disseminated Coccidiomycosis occurs in approximately 1% of infected individuals with its most severe form being meningitis [4]. We report a case of *C. posadasii* meningoencephalitis in a 42-year-old male who returned to Canada after spending time working in New Mexico.

## Case presentation

A previously healthy 42-year-old Caucasian male presented to the emergency department of a tertiary care center complaining of a 3-week history of headache, malaise and low-grade fevers. He returned to Canada after spending 28 days living in a trailer 100 km outside of Hobbs, New Mexico, working on the oil rigs. He recalled exposure to live and dead rats in his trailer as well as multiple insect bites. His travel history was significant for a trip to Panama two years prior with his wife and children. He denied any ingestion of raw meats, raw seafood or unpasteurized dairy.

The patient had developed sudden onset fever, myalgias and severe headache while he was in New Mexico. His headache was persistent with waxing and waning features accompanied by photophobia/phonophobia, presyncope and nausea. He returned home 15 days after symptoms began. On day 21, he presented to the emergency department complaining of non-resolving headaches, fevers and vomiting.

**Fig. 1:**
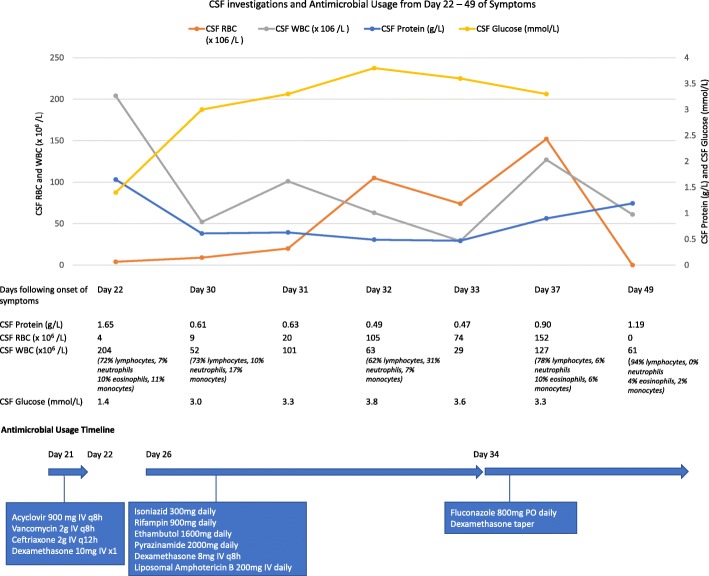
Chronologic representation of serial CSF measurements obtained from lumbar puncture or external ventricular drainage (EVD) catheter with CSF RBC, CSF WBC count on the left axis and CSF protein, CSF glucose on the right axis. Antimicrobials used during this timeline are documented in association to the CSF values

On admission to hospital, he was febrile at 38.0 °C and diaphoretic. He had difficulty with complex cognitive tasks such as word finding and recall [mini mental status exam (MMSE) of 24/30]. He had no rash, no focal neurologic abnormalities and no signs of meningismus. The remainder of his physical examination was unremarkable. His CBC, electrolytes, creatinine and CRP were all within normal range. Imaging included a normal computed tomography (CT) head with IV contrast and a normal CT of his chest with no lymphadenopathy. Empirically, he was treated for both viral and bacterial meningitis (Fig. 1). A lumbar puncture (LP) was performed and all antimicrobials were discontinued following the cerebrospinal fluid (CSF) results (Fig. 1).

**Fig. 2:**
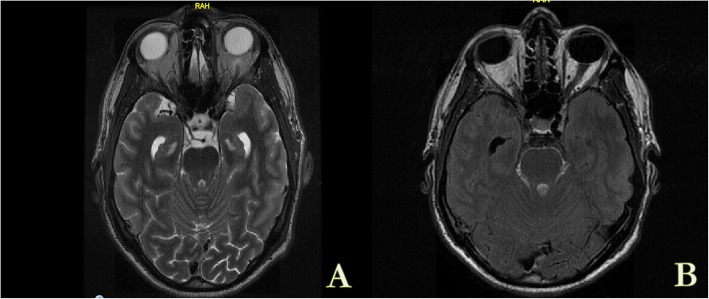
MRI Brain with diffuse smooth, leptomeningeal enhancement, predominantly within the basal cisterns and surrounding brainstem associated with cranial nerve enhancement most pronounced along the pre-chiasmatic optic nerves and 3rd cranial nerves with interval development of hydrocephalus noted. **a** Axial T2 Flair. **b** Axial T1 Flair post Gadolinium enhancement

Following initial resolution of fevers and improvement in his headache, on day 26 of symptoms the patient began to deteriorate. He developed progressive confusion and subsequent decreasing level of consciousness. A magnetic resonance imaging (MRI) of his brain was performed (Fig. 2). Based on the MRI changes, empiric meningitis treatment to cover both tuberculous (TB) (isoniazid 300 mg daily, rifampin 900 mg daily, ethambutol 1600 mg daily, pyrazinamide 2000 mg daily, dexamethasone 8 mg IV q8h) and fungal etiologies were initiated (liposomal amphotericin B 200 mg IV daily) (Fig. 1). An external ventricular drainage (EVD) catheter was placed on day 30.

The patient’s serum and CSF were tested for the presence of IgM and IgG against Coccidoides using a commercial enzyme immunoassay (Premier®). The serum IgM was negative, IgG positive and CSF was positive for both IgG and IgM. A polymerase chain reaction (PCR) using pan-fungus primers targeting ribosomal RNA genes and internal transcribed space (ITS) sequences, was positive for *Coccidioides posadasii* (Table 1)*.*

**Table 1:** Summary of investigations divided by date and location of collection. Appropriate anti-fungal therapy was started on day 27 of symptoms

Sample	Test	Result
Blood		
Day 22	Antibodies against arbovirus antigens.	Negative
Day 28	*Bartonella henselae* IgG	Negative
	Blastomyces Immunodiffusion IgG	Negative
	**Coccidioidal IgG**	**Positive**
	Coccidioidal IgM	Negative
	Coccidiodal Immunodiffusion IgG	Negative
	HIV 1 and HIV 2 antibody	Negative
	Toxoplasma IgG and IgM	Negative
	Syphilis antibody	Negative
Day 29	Mycobacterial culture	Negative
Day 30	Flow cytometry for lymphoma	Negative
	**Coccidiodal PCR**	**Positive**
	(pan-fungus primers targeting ribosomal RNA genes and ITS sequences)	***(Coccidiodes posadasii)***
Cerebrospinal fluid		
Day 22	Antibodies against arbovirus antigens	Negative
	Enterovirus RT-PCR	Negative
	Herpes simplex virus PCR	Negative
	Varicella zoster virus PCR	Negative
	West Nile virus RT-PCR	Negative
	Mycobacteria (AFB) GeneXpert	Negative
Day 30	Cryptococcal antigen	Negative
	Mycobacteria (AFB) smear and culture	Negative
	**Coccidioidal IgG**	**Positive**
	**Coccidioidal IgM**	**Positive**
	Coccidiodal Immunodiffusion IgG	Negative
Stool		
Day 30	Mycobacteria (AFB) Smear and Culture	Negative

The patient defervesced and experienced rapid clinical improvement. On day 34 of symptoms the EVD was removed. The anti-TB therapy was discontinued. His course was complicated by acute kidney injury thought to be associated with liposomal amphotericin B and therapy was changed to high dose fluconazole 800 mg PO daily. He was discharged home on day 37 of symptoms.

He continued to have improvement of his cognitive status and his headaches resolved. Repeat MRI revealed resolving leptomeningeal enhancement. Indefinite Fluconazole therapy was initiated. His course was complicated by limb and trunk alopecia related to his fluconazole, which resolved after decreasing his fluconazole dose to 400 mg PO daily. At 18 months following his initial diagnosis he continues to have significant short-term memory deficits, has not returned to his full baseline functional status and has been unable to return to work.

## Discussion and conclusions

Involvement of the CNS can occur in up to 50% of patients with disseminated coccidiomycosis occurring within weeks to months of primary infection [11]. The most common presentation is basilar meningitis, which may be complicated by hydrocephalus and vascular infarcts [4, 12]. The consequence of unrecognized CNS coccidiomycosis can be devastating and therefore early recognition and treatment is imperative to minimize mortality and morbidity [4].

In a review of 71 cases of coccidioidal meningitis (CM), males accounted for over two thirds of cases, 42% were immunocompromised and 45% described a preceding illness suggestive of pulmonary coccidiomycosis [12]. The most common symptom of CM is headache, other symptoms include fever, neck stiffness, nausea and vomiting [3, 11]. Rarely patients can experience change in personality, cognitive abnormalities, decreased level of consciousness and focal neurologic symptoms. In 50% of cases an MRI will show no abnormalities [2, 3, 15].

In CM, CSF results reveal a lymphocytic pleocytosis (however in early disease may have a neutrophilic predominance), hypoglycorrhachia and elevated protein [2, 3, 12]. It is important to distinguish between CM and other causes of chronic meningitis, most notably TB meningitis. CSF eosinophilia is seen in 10% of patients with CM but is a very rare finding in TB meningitis [7, 12–14]. CSF eosinophilia can also be seen with parasitic etiologies (*Angiostrongylus cantonensis*, *Baylisascaris procyonis* and *Gnathostoma spinigerum*), non-parasitic infections (lymphocytic choriomeningitis virus), and non-infectious etiologies (hematologic disorders, drug reactions or shunt malfunctions) [13–15]. Other etiologies considered in this case included; leptomeningeal lymphoma, neurosyphilis, lyme neuroborreliosis, HIV meningoencephalitis, toxoplasma cerebritis and West Nile encephalitis.

A CSF culture positive for *Coccidioides* or a CSF Coccidioidal IgG antibody is virtually diagnostic for CM. The most reliable tests are serology with ELISA for initial screening followed by immunodiffusion tests for IgM and IgG and complement fixation for IgG. Immunodiffusion or complement fixation for IgG is very highly specific, however the sensitivity is 30–60% [2, 3]. If results are inconclusive and suspicion high, PCR of CSF can help in the diagnosis of *Coccidioides* [7, 16]. The use of 1,3-Beta-D-Glucan testing in CSF may be a useful test for ruling out CM, particularly in immunocompromised patients who may have delayed antibody production. In a 2016 study by Stevens et al., the use of CSF 1,3-Beta-D-Glucan in diagnosis of CM was investigated in 37 patients revealing a test sensitivity of 96% and specificity of 82% [17].

CM has a mortality of 90% in one year and 100% in two years if left untreated [17]. The advent of amphotericin B deoxycholate and ability for intrathecal administration reduced mortality to 30% [11]. The gold standard of treatment is now fluconazole. Infectious Diseases Society of America (IDSA) guidelines recommend lifelong ‘azole’ therapy for CM as they are fungistatic agents with rates of relapse after discontinuation of therapy of nearly 80% [2, 18]. Treatment dosing ranges from 400 to 1200 mg daily, but 800–1200 mg daily is preferred given a lower risk of disease relapse [2, 3, 11]. Alopecia, as seen with this case, is a rare side effect of fluconazole associated with doses of greater then 400 mg daily for ≥2 months. The alopecia is reversible with discontinuation of fluconazole or 50% reduction in dosage [19].

Alternative therapeutic options include itraconazole, voriconazole and posaconazole, however CNS penetration with these antifungals is less. Intrathecal amphotericin B deoxycholate is now only used as a rescue agent in those failing “azole” therapy due to significant administration risks and side effects [3, 11]. The benefit of using adjuvant glucocorticoids in CM is not well studied nor stated in IDSA guidelines, however steroids are used by most experts treating CM [2, 3]. Unfortunately, despite adequate and prompt therapy, many patients will experience complications of hydrocephalus, vascular infarction, cerebral vasculitis, cranial neuropathy or arachnoiditis that can result in long lasting cognitive complications varying in severity [2, 3].

Travel to endemic regions can result in the acquisition of Coccidiomycosis for both immunocompetent and immunosuppressed individuals. The morbidity and mortality of CM is devastating, and the prognosis can depend on early recognition and treatment. Physicians in non-endemic regions must be aware of CM along with its risk factors, disease presentation, complications, diagnostics and treatment.

## Data Availability

Data sharing is not applicable to this article as no datasets were generated or analyzed during the current study.

## Abbreviations

CBC: Complete blood count
CM: Coccidioidal meningitis
CRP: C-reactive protein
CSF: Cerebrospinal fluid
CT: Computed tomography
ELISA: Enzyme-linked immunosorbent assay
EVD: External ventricular drainage catheter
HIV: Human immunodeficiency virus
IDSA: Infectious Diseases Society of America
ITS: Internal transcribed space
LP: Lumbar puncture
MMSE: Mini mental status exam
MRI: Magnetic resonance imaging
PCR: Polymerase chain reaction
TB: Tuberculous
